# 3D Deformation Patterns of S Shaped Elastic Rods as a Pathogenesis Model for Spinal Deformity in Adolescent Idiopathic Scoliosis

**DOI:** 10.1038/s41598-019-53068-7

**Published:** 2019-11-11

**Authors:** Saba Pasha

**Affiliations:** 1 Department of Orthopedic Surgery, Perelman School of Medicine, University of Pennsylvania, Philadelphia, PA USA; 2 Division of Orthopedic Surgery, The Children’s Hospital of Philadelphia, Philadelphia, PA USA

**Keywords:** Paediatric research, Pathogenesis, Biomedical engineering

## Abstract

Adolescent idiopathic scoliosis (AIS) is a three-dimensional (3D) deformity of the spinal column in pediatric population. The primary cause of scoliosis remains unknown. The lack of such understanding has hampered development of effective preventive methods for management of this disease. A long-held assumption in pathogenesis of AIS is that the upright spine in human plays an important role in induction of scoliosis. Here, the variations in the sagittal curve of the scoliotic and non-scoliotic pediatric spines were used to study whether specific sagittal curves, under physiological loadings, are prone to 3D deformation leading to scoliosis. To this end, finite element models of the S shaped elastic rods, which their curves were derived from the radiographs of 129 sagittal spinal curves of adolescents with and without scoliosis, were generated. Using the mechanics of deformation in elastic rods, this study showed that the 3D deformation patterns of the two-dimensional S shaped slender elastic rods mimics the 3D patterns of the spinal deformity in AIS patients with the same S shaped sagittal spinal curve. On the other hand, the rods representing the non-scoliotic sagittal spinal curves, under the same mechanical loading, did not twist thus did not lead to a 3D deformation. This study provided strong evidence that the shape of the sagittal profile in individuals can be a leading cause of the 3D spinal deformity as is observed in the AIS population.

## Introduction

Adolescent idiopathic scoliosis (AIS) manifests as a three-dimensional (3D) deformity of the spinal column that occurs around puberty. Several factors such as genetics^1,2^, biomechanics^3^, morphology of the vertebral bones, intervertebral disc, and muscle physiology^4–9^, hormones^10,11^, environmental factors^12,13^, and neural system deficiencies^14,15^ are considered as the underlying cause of the spinal deformity in AIS. While each of these factors explained part of the pathogenesis of this disease, none have proved to be its main or primary cause. As experimental studies suggest, the role of the mechanical loading of the upright spine in pathogenesis of the disease cannot be ignored^16–21^.

The unique shape of the human spine has been hypothesized to relate to the induction of scoliosis^3^. The dorsal loading of the upright spine in human was proposed to result in spinal instability and has been suggested as the biomechanical causation of scoliosis^22^. A shift in the position of the center of mass around puberty results in asymmetric loading of the thorax^23^. This causes rotation of the spinal segments away from the sagittal plane which in turn results in an increased differentiated loading of the anterior and posterior aspects of the vertebrae, causing asymmetric growth and bone deformation under the Hueter-Volkmann law^24,25^. While the mechanism of deformity *progression* in AIS is widely accepted, the underlying mechanisms that trigger instability in the spine and excessive rotation, leading to scoliosis in 2–4% of the pediatric population, are not well explained.

**Figure 1:**
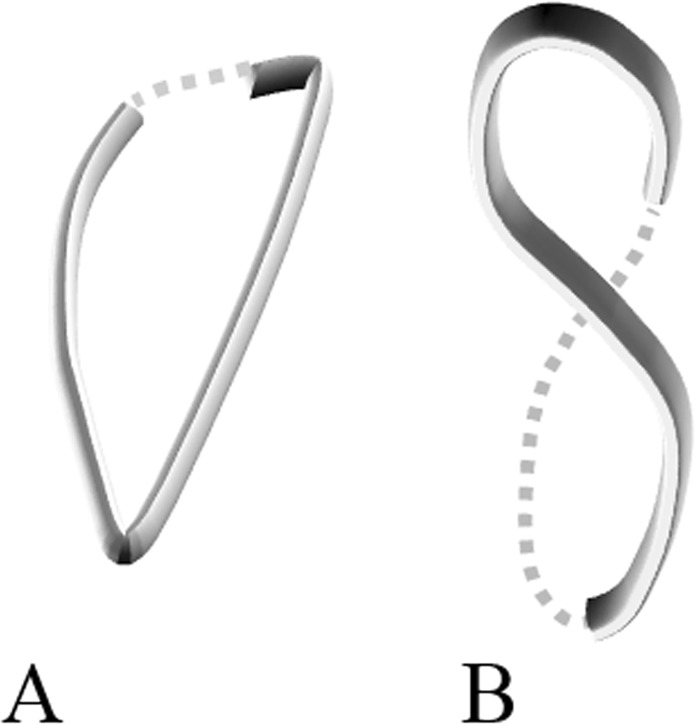
Two different axial projections of the scoliotic spines^33^ (**A**) *V shaped* projection that can be extrapolated to a closed loop and (**B**) *S shaped* projection that can be extrapolated to a twisted loop (lemniscate).

The mechanics of deformation in elastic rods are explored experimentally and analytically^26–29^. These concepts have been successfully explained the physics of various topological transformation in DNA and proteins (*e.g*., from a loop to a twisted loop)^30–32^. In a previous topological classification of the spinal curves in AIS patients^33,34^, two distinct axial deformation patterns of the scoliotic spines were observed: if a non-scoliotic spine projects into a straight line from the top view (axial view), the scoliotic spine has either a *V shaped* or a *S shaped* projection^34^. The *V shaped* projection can be conceived as a loop (by connecting the two ends) whereas the *S shaped* projection presents a twisted loop or a lemniscate shape. Figure 1 demonstrates these two different projection patterns. As these two axial projections (Fig. 1) were associated with different sagittal curves in the scoliotic classification^34^, the question was raised that whether the variation in the sagittal curves of the pediatric spine dictates the patterns of spinal deformation and can be the leading cause of the 3D spinal deformity *i.e*. scoliosis.

In this study, finite element analysis (FEA) of two-dimensional (2D) *S shaped* elastic rods was conducted to determine the deformation patterns of the rods as a function of variations in the 2D curve geometry, while the mechanical loading and material properties were the same across the models. The geometry of the 2D *S shaped* rods were determined from the two-view spinal X-rays of 126 right thoracic AIS patients and three age- and sex-matched non-scoliotic adolescents. It was hypothesized that the patterns of 3D deformation of 2D *S shaped* elastic rods (spine sagittal profiles) relate to the 3D patterns of the spinal deformity in the AIS patients with the same sagittal curves.

## Results

**Figure 2:**
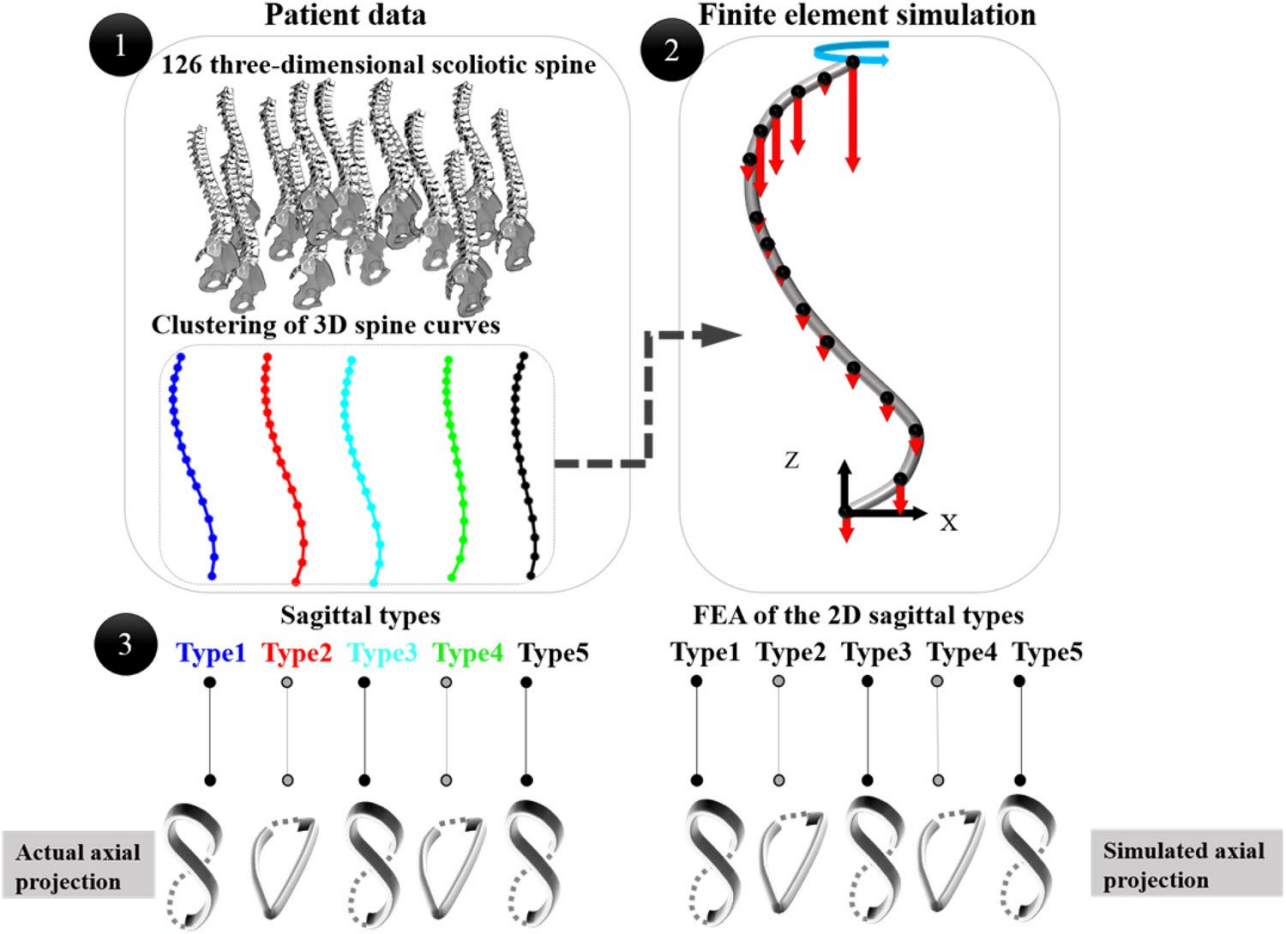
The schematic of the study workflow: 1- Clustering of 126 right thoracic AIS and identifying the sagittal subtypes. 2- Finite element analysis (FEA) of the curved elastic rod – the geometry of the rods were determined from the AIS sagittal subtypes. The red arrows show the magnitude of the gravitational load at each vertebral level (Supplementary Materials-Table 1S). The blue arrow shows the direction of the torsion moment. 3- Comparison between the patterns of 3D deformation of the spine in AIS subtypes and the curved (*S shaped)* elastic rods with the same 2D curvature as the AIS sagittal subtypes.

The study’s workflow is shown in Fig. 2. The study steps include: 1- classification of 126 scoliotic 3D spinal curves^34,35^, 2- developing the finite element models of the curved elastic rods using the *S shaped* sagittal spinal curve of the scoliotic cluster centers and the average of three non-scoliotic spines. 3- Comparing the patterns of deformation between the actual spinal deformity in the patient subtypes and the simulated curved rods deformations.

### Classification and sagittal subtypes

**Figure 3:**
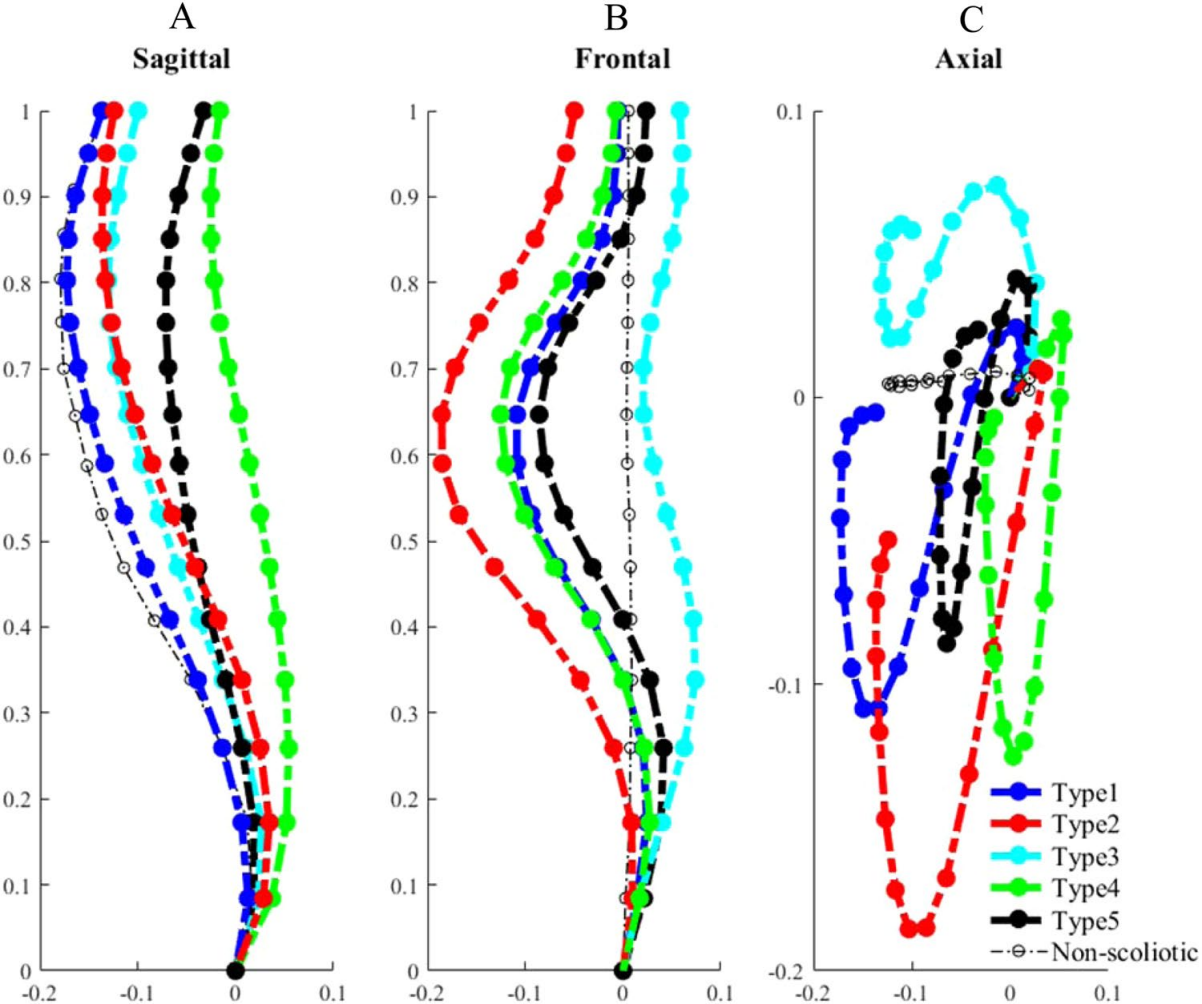
Five right thoracic scoliotic subtypes and the average of non-scoliotic spinal profiles in (**A**) Sagittal, (**B**) Frontal, (**C**) Axial views. The X, Y, Z axes show unite-less normalized distances.

Figure 3 shows the five subtypes of the 126 right thoracic AIS patients resulted from the clustering of the 3D spinal curves: Type 1: n = 30, Type 2: n = 29, Type 3: n = 18, Type 4: n = 26, and Type 5: n = 23. The five subtypes’ sagittal, frontal, and axial views of the spinal curves are shown in Fig. 3A–C, respectively. The average of the non-scoliotic spinal curves is also superimposed on Fig. 3. The axial and frontal projections of the non-scoliotic spine were close to a straight line (Fig. 3B,C).

The five scoliotic subtypes were divided into two subgroups based on the patterns of axial projections: Group I included Types 2 and 4 that have a loop shaped axial projection (Fig. 3C). In these patients the kyphotic (concave) segment of the curve was shorter than the lordotic (convex) section (6 ± 2 vertebrae versus 11 ± 2) and the inflection point of the curve moves to the mid thoracic section (see Methods section). Group II included Types 1, 3, and 5 that have a lemniscate axial projection (Fig. 3C). In these patients the kyphotic part of the curve was longer than the lordotic section (12 ± 2 versus 5 ± 2 vertebrae) and the inflection point of the sagittal curve moves to the lower thoracic/ upper lumbar area. In the non-scoliotic cohort the kyphotic section is longer than the lordotic section (12 ± 2 versus 4 ± 1 vertebrae), but the inflection point is slightly lower compared to the position of the inflection point in Group II.

### Finite element analysis and 3D deformation

**Figure 4:**
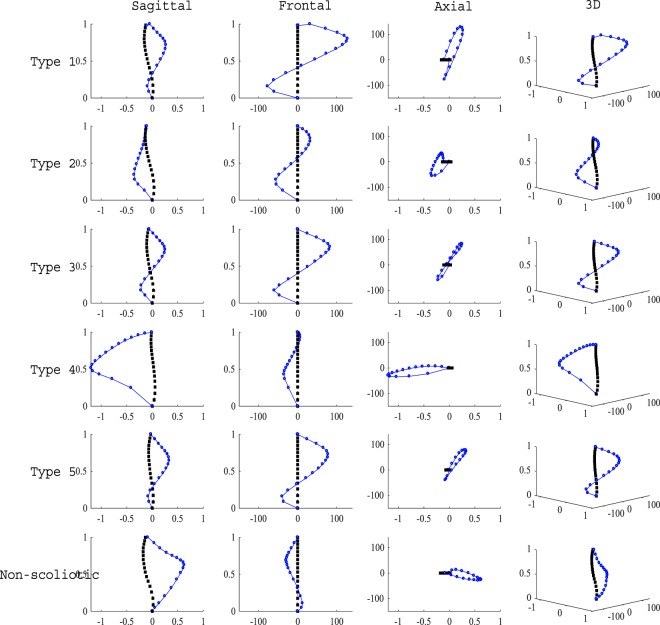
The initial (black line) and final curvature (blue line) of the elastic rods representing the five scoliotic sagittal subtypes and non-scoliotic spine under gravitational loading and torsion in frontal, sagittal, axial, and 3D views. The axes show the isotropic normalized lengths in the Z-direction for both initial and deformed curves.

Figure 4 shows the results of the FEA of the five sagittal subtypes and the non-scoliotic spines. The final shape of the deformed rod (blue dotted line) was superimposed on the original shape of the rods (black dotted line), in the frontal, sagittal, axial, and 3D views (Fig. 4). All rods developed two curves in the frontal planes under the aforementioned loading (Fig. 2). The axial shape of the deformed rods with the geometry of the Types 2 and 4 scoliotic subtypes (Group I) was loop shaped. The axial shape of the deformed rods with the geometry of the Types 1, 3, and 5 scoliotic subtypes (Group II) were lemniscate. The axial shape of the “non-scoliotic” deformed elastic rod was loop shaped but deformed in the opposite direction of the axial projection of the Group I curves.

**Figure 5:**
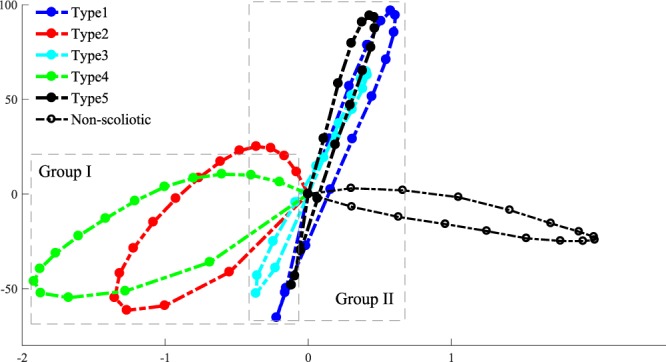
The superimposed axial projection of the normalized 3D deformation of the *S shaped* rods. The X and Y axes show unite-less normalized distances.

The axial deformations (differences between the final and initial positions of each point on the rod) of the rods representing the sagittal subtypes and non-scoliotic spines are superimposed in Fig. 5 to allow a better comparison between the deformation patterns of the Group I AIS (Types 2 and 4), Group II AIS (Types 1, 3, and 5), and the average of the three non-scoliotic adolescents. The deformations are normalized to the maximum deformation in the Z direction. Group I rods showed a looped shaped axial deformation (also Supplementary Materials Fig. 3S-A) and Group II rods showed a lemniscate axial deformation (also Supplementary Materials Fig. 3S-B). The axial deformation of the non-scoliotic rod was distinctively different from Group I and Group II (also Supplementary Materials Fig. 3S-C). The ratio of the average displacement in the X direction to the Y direction was: Type1 = 0.004, Type2 = 0.05, Type3 = 0.004, Type4 = 0.06, Type5 = 0.006, and non-scoliotic = −0.01.

**Figure 6:**
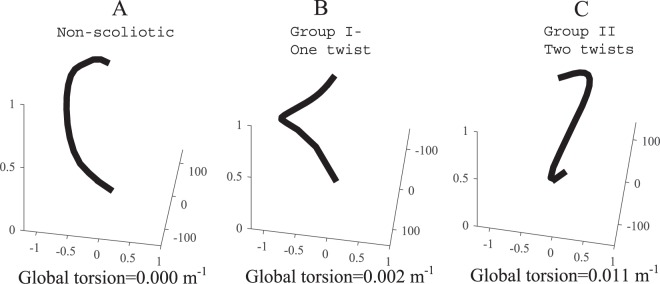
Normalized average 3D deformation of the elastic rods in the “non-scoliotic”, Group I, and Group II rods. The X, Y, Z axes show unit-less normalized distances. The global torsion for each curve is shown^65^.

The average deformation shape of the rods in non-scoliotic, Group I, and Group II are shown in Fig. 6. In the non-scoliotic curve, the rod deformation remained in one plane and no twist in the rod was observed (Fig. 6A). The curved rods in Group I (Types 2 and 4) showed one twist in the (Fig. 6B). Rods in Group II (Types 1, 3, 5 models) showed two twists (Fig. 6C). The global torsion of the non-scoliotic deformed curved (Fig. 6A), average Group I deformed curve (Fig. 6B), and average Group II deformed curve (Fig. 6C) were −3.4e-4(m^−1^), 0.0015(m^−1^), and 0.0112(m^−1^), respectively.

The 3D deformation of the additional *S shaped* rods, resulted from interpolating the sagittal curves in Group I (Types 2 and 4), sagittal curves in Group II (Types 1, 3, and 5), and the three non-scoliotic curves are presented in the Supplementary Materials.

### Pattern correlation between the scoliotic subtypes and non-scoliotic deformation of the *S shaped* rods

The results of the pattern correlation of the 3D deformation (X, Y, Z coordinates) of the rods between Types 1–5 and non-scoliotic 3D deformations are shown in Table 1. The 3D deformations in Group I were strongly correlated; Types 2 and 4, r = 0.89, 95%CI [0.71–0.88], p = 0.00. The 3D deformations in Group II were strongly correlated; Types 1 and 3 and Types 1 and 5, r = 0.99, 95%CI [0.99–0.99], p = 0.00, and Types 3 and 5 r = 0.98, 95%CI [0.96–0.98], p = 0.00. The correlation between the 3D deformation in Groups I and II was non-significant (Types 1 and 4, Types 3 and 4, and Types 4 and 5) or moderately correlated (Types 1 and 2, Types 2 and 3, and Types 2 and 5) (Table 1). 3D deformation of the AIS clusters were significantly correlated to the 3D deformation of the non-scoliotic spines, however the correlation was not as strong as the within AIS groups correlations (Table 1).

**Table 1:** Pattern correlation of the 3D rod deformation between the scoliotic subtypes and the non-scoliotic group.

1	2	3	4	5	Non-scoliotic	Types
1	0.72 [0.55–0.83], p = 0.00	0.99 [0.99–0.99], p = 0.00	0.18 [−0.09, 0.43], p = 0.81	0.99 [0.99–0.99], p = 0.00	0.64 [0.61, 0.89], p = 0.00	1
	1	0.73 [0.69–0.89], p = 0.00	0.89 [0.71–0.88], p = 0.00	0.69 [0.51–0.81], p = 0.00	0.73 [0.62–0.91], p = 0.00	2
		1	0.33 [0.06–0.56], p = 0.83	0.98 [0.96–0.98], p = 0.00	0.70 [0.61,0.88], p = 0.00	3
			1	0.15 [−0.13,0.41], p = 0.72	0.65 [0.60,0.85], p = 0.00	4
				1	0.61 [0.49,0.80], p = 0.00	5
					1	Non-scoliotic

## Discussion

The pathogenesis of the spinal deformity development in AIS is not well understood. Here, concepts in mechanics of elastic rods were used to show that the variations in the sagittal curve relate to the 3D deformation patterns of the spine in AIS. Finite element models of the 2D curved elastic rods, representing different sagittal types in 126 AIS patients^34^ and three non-scoliotic adolescents, showed that deformation pattern of the curved elastic rods changes as a function of the rod’s curvature and associates with the patterns of 3D spinal deformity in AIS patients with the same sagittal curves. This finding provided evidence that the sagittal curve of the spine is a leading cause of spinal deformity in AIS.

Several biomechanical factors have been related to the mechanism of the spinal deformity development in AIS. Slender vertebrae and discs in AIS patients, compared to the non-scoliotic age-matched adolescents, is believed to increase the spinal flexibility and lead to spinal deformity in AIS^36^. The sagittal profile in AIS patients was reported to be more posteriorly inclined which increases the posteriorly directed shear force in AIS compared to the age-matched non-scoliotic adolescents contributing to the spinal instbility^37^. Morphological parameters of the vertebrae and discs were related to the progression rate of scoliosis^38^. It has been also suggested that the mechanical loading of the upright spine can be the primary cause of scoliosis^22^. The axial loading of the spine is higher in quadrupeds than bipedal’s spines^39^. In bipedals a less axially supported spine, which probably contributes to easier upright locomotion^40,41^, may be impacted by other spinal loadings e.g. shear and make the spine less stable^22^. The current analysis showed, in an elastic rod, without considering the differences in the spinal components (discs, vertebrae)^36,38^ and without generalizing the vertebral alignment^37^, and under the same external loadings, some sagittal subtypes do not twist and deform in only one plane (Fig. 6A) whereas other sagittal curve types are prone to scoliotic-like 3D deformation with one or two twists in the spine (Fig. 6B,C). The 2D shape of the rod changed the acting forces and moments on the rod resulting in variation in the deformation patterns, underlining the role of the spinal sagittal curve in development of spinal deformity.

The classification of the 3D centerlines in this study resulted in identifying a clear axial classification of right thoracic AIS: loop and Lemniscate shapes (previously presented as *V* and *S* shaped, respectively)^34^ (Fig. 1). These shapes are also shown in other biological and mechanical systems^28,30–32^. We also showed these two axial curve patterns, within the right thoracic cohort, can guide the fusion level as they determine the location of the changes in the axial rotation of the vertebrae thus the number of true 3D spinal curves^42^. These findings may allude to the fact that the patterns of deformation of the spine in the transverse view, as determined in this study, can explain important differences between the scoliotic curves and can be used as an axial classification of the scoliotic curve.

3D elastic deformation has been observed in many natural and physiological systems such as proteins and DNAs^30–32^. A looped DNA can twist and transform to a lemniscate shape as a function of bending rigidity of the strands when the system’s energy attains a critical level^32,43,44^. The fundamentals of these deformations are explained analytically using Kirchhoff’s theory of elastic rods^44,45^. In analytical examination of the spinal deformity in AIS, mechanical phenomenon such as 2D buckling of the rods have been used to explain the mechanics of spinal deformation previously^36,38,46–53^. However, considering the 3D deformation of the spine in AIS, the mechanics of 2D buckling fails to explain essential aspects of the curve progression. By taking into account the sagittal curve of the spine, *both* bending and compression stresses apply on the spine as a result of the gravitation load. Here, a torsional moment also was included to simulate the previously reported shift in the center of mass at the onset of curve development in AIS patients^23^. This torsion was essential to break the symmetry of the system and initiate the 3D deformation. The result of the current simulation shifts the current beliefs about the mechanism of the spinal deformation in AIS from 2D buckling to 3D elastic deformation under bending and twisting. Understanding the mechanism of spinal deformity development in AIS is the first step in efficient management of the disease that aims to prevent the deformity development or reverse the deformation process.

Modeling the spine as elastic rods assists with understanding the intra-patient differences, which can be useful in clinical management of the scoliosis. As it is shown before, different deformation patterns in elastic rods can be observed by changing the mechanical loading, material stiffness, boundary conditions, or geometry of the rod^28,29^. It has been shown that a gradual increase in the twist and torsional energy can change the coiling pattern resulting in a twist in the rod^28^. This phenomenon can explain the differences between the curve patterns in Group I and Group II. Group I patients present with one twist in the curve, smaller torsion, and larger lateral deviation as opposed to the Group II patients with two twists, smaller lateral deviation (coiling radius), and high torsion^34^ (Fig. 6). As it was shown previously, due to the differences between Group I (Type 2 and 4) and Group II (Types 1, 3, and 5), the patients’ response to the spinal fusion surgery may vary, thus different surgical fusion lengths should be selected for these two groups: Longer fusion in Group I patients is required to stabilize the longer curve as opposed to the patients in Group II where spontaneous correction of the second curve can be expected as a response to the primary curve derotation and correction^42^. By showing the relationship between the sagittal curve types and deformation patterns in elastic rods, the importance of the spinal sagittal curve correction in both conservative and surgical treatments of AIS were emphasized which was in line with the previous observations^35,54–57^. Reversing the 3D spinal deformation by derotation and frontal translation of the spine in AIS does not adequately address the condition as here we showed that variation in the sagittal profile could initiate the deformity and is the underlying cause of the deformity. As the variation in sagittal profiles is an important factor in 3D deformation of the spine (Fig. 6), it stands to reason that adjustments in the sagittal plane as part of the curve correction process, both conservatively and surgically, are critical.

Quantitative differences between the simulated deformations of the curved elastic rods and the deformation of the spine in patient groups with the same sagittal curve were expected. Due to the simplifications of the model (*e.g*., the isotropic homogeneous material properties, ignoring the differential stiffness between the upper and lower sections of the rod (thoracic and lumbar), assuming a zero angle at the L5 (sacral slope)) quantitative differences in the simulated rods and actual 3D spinal deformation were observed; yet *patterns* of deformations were similar. Moreover, T1 was fixed in the axial plane while in reality both frontal and sagittal imbalances are shown as part of the curve progression mechanism and the postural compensation of the trunk ^8,58^. This study purposefully used a reduced order model of the spine to test the theoretical role of the spinal sagittal shape in 3D scoliotic deformation without including any other assumption on intra-patient variations.

The mechanics of the 3D deformation of elastic rods showed that the 3D deformation patterns of the spine can be a characteristic of the *S shaped* curvature of the sagittal spine. However, it is important to notice that this finding does not refute the possibility of forming such sagittal profiles or variation in the mechanical loading (deformation) in individuals as a result of variations in the discs and ligaments material properties or width and height of the vertebral body. Genetics can tie into the development of various sagittal alignment as it may impact the discs, ligaments, and muscles biomechanics, growth rate, or simply the postural differences between individuals. Both rapid growth of the spine and rapid rise of estrogen level during puberty^52,53,59,60^, and the link between the estrogen and increased ligament laxity^61,62^ can be linked to the onset of the spinal deformation at that age particularly in the female patients. The current analysis only investigated the role of the sagittal profile in development of 3D spinal deformities. The factors leading to variations in the loading characteristics of the sagittal profile, related to the geometry, growth rate, and mechanical properties of the spine, remain to be explored. Finally, in the current study only right thoracic curves, the most common type of AIS, were included. Future studies that include other curve types are warranted.

In conclusion, the finding of this study reduced the question of why scoliosis happens to why variation in the sagittal spinal profile occurs in the pediatric population. With large dataset on the pediatric population before the onset of the spinal deformity development, the current analysis can be used as a risk assessment tool to predict the risk of spinal deformity development in the pediatric population. This can open a new line of research that aims to prevent the spinal deformity development by determining the spinal loading patterns (the sagittal alignment and flexibility of the immature spine) at a higher risk of 3D deformation in *pre-scoliotic* pediatric population. Conservative methods to protect the spine during this phase can be explored. This new approach can introduce a changing paradigm in the clinical care of idiopathic scoliosis by shifting the clinical care from the deformity correction to prevention in this patient population.

## Methods

### Classification and identifying unique sagittal profiles in AIS patients

The institutional review board (IRB) at the Children’s Hospital of Philadelphia approved all the study procedures as detailed in the method section. The research process was performed in accordance with the relevant guidelines and regulations underlined in the approval. As the study was performed retrospectively, a waiver of consent/parental agreement was granted by the IRB.

A total of 126 right thoracic AIS patients were included retrospectively. The inclusion criteria were adolescent between 12–16 years old, a main thoracic curve (most common AIS curve type) exceeding 20° Cobb angle with an apex above T11-T12 intervertebral disc, no previous spinal surgery, no neuromuscular disorders, and no musculoskeletal conditions other than scoliosis. Three non-scoliotic adolescents were included as the control cohort. The non-scoliotic subjects were verified by both clinical examination and two-view radiographs of the spine and did not develop scoliosis at the follow-ups after growth spurt.

3D reconstructions of the orthogonal spinal radiographs were generated for all the subjects using the method described in Humbert *et al*.^63^. A previously validated algorithm was used to extract the centroid of the vertebral bodies^64^. A K-mean classification, with a prior knowledge of the number of clusters^33^, was used to classify the isotropically normalized 3D spline (X, Y, Z coordinates) resulted from connecting the vertebral bodies centroids^35^. The sagittal profiles of the non-scoliotic spines were also isotropically normalized to the unit height.

### Data preparation

The average sagittal profiles of each AIS cluster was used to create the geometry of the 2D curved (*S shaped)* elastic rods in the finite element models. The sagittal profiles of the clusters were grouped based on their axial projection patterns; Group I: loop shaped projection and Group II: twisted loop or lemniscate projection (Fig. 1). Additional sagittal profiles were generated by interpolating between the sagittal profiles of the clusters in Group I and between the sagittal profiles of the clusters in Group II. This step was added to include additional *S shaped* curves and test the sensitivity of the 3D deformation patterns of the *S shaped* rods to variations in the sagittal curves. The global torsion of the average deformed curves in the non-scoliotic groups, Group I, and Group II were calculated by the method described in^65^ Based on the sagittal view, the number of vertebrae was counted in the two sections of the spine with different curvatures i.e. spinal convexity and concavity in the sagittal plane. The point at which the sign of the curvature changed or the center of the section over which the sign of the curvature changed was considered as the inflection point.

### Finite element models

2D *S shaped* elastic rods with unit length, circular cross section with a radius of 1e-3, Young modulus of 1000 PA, and a Poisson ratio of 0.3, with isotropic material properties were generated. A small torsion around the Z-axis, +1e-4 Pa, was applied to simulate the asymmetric trunk weight distribution^23^. The X, Y, Z displacement of the rod at L5 level were fixed. The X and Y displacements at T1 were fixed but the vertical (Z direction) displacement was allowed. The gravity was applied at T1-L5 vertebral centers according to the previously validated experimental data for FEA of the spine^66–69^ (Table 1.S- Supplementary Material). The FEA was performed for the rods with the sagittal geometry of the scoliotic subtypes and the interpolated sagittal curves, and for the average of the non-scoliotic curves and the individual non-scoliotic spines. The 3D deformation was normalized to the maximum deformation in the Z direction.

### Pattern correlation of the deformed rods

The 3D shapes of the deformed 2D *S shaped* rods were compared between the models in Group I and Group II as well as between the non-scoliotic model and the two AIS groups. 3D pattern correlation related the X, Y, Z coordinates of the deformed rods between the models by calculating the covariance matrix of the 3D deformation patterns to compare the inter- and intra-group correlations between the 3D deformations.

## Supplementary information

supplemetary information

## Data Availability

Non-patient related data that support the findings of this study are available from the author upon reasonable request.

## Code availability

The executable code is available from the author upon reasonable request for non-commercial purposes only.
