# Supplementary material for: 3D Deformation Patterns of S Shaped Elastic Rods as a Pathogenesis Model for Spinal Deformity in Adolescent Idiopathic Scoliosis
Source: Sci Rep. 2019 Nov 11;9:16485. doi: 10.1038/s41598-019-53068-7 (PMC6848095; doi:10.1038/s41598-019-53068-7)
Supplement: Supplementary file 1 — supplemetary information [file 41598_2019_53068_MOESM1_ESM.docx]

**Supplementary materials**

**3D Deformation Patterns of S Shaped Elastic Rods as a Pathogenesis Model for Spinal Deformity in Adolescent Idiopathic Scoliosis**

Saba Pasha*, PhD

Department of Orthopedic Surgery, Perelman School of Medicine, University of Pennsylvania, Philadelphia, PA, USA

Division of Orthopedic Surgery, The Children's Hospital of Philadelphia, Philadelphia, PA, USA

*Correspondence to pashas@pennmedicine.upenn.edu

**Gravitational load modeling**

The detail of the gravitation load at each vertebral level is presented in table 1S. This data was adapted from Pearsall et al. and Liu et al.^1,2^ and were used previously in finite element analysis of the scoliotic spine^3,4^. The graviaional force was calculated for unit body mass.

Table 1S: Application of the gravitation load in the finite element model. The vertical loads were applied at the center of T1-L5 vertebrae. Gravity =-9.8 N/m^2^

| Vertebral level | % body mass |
| --- | --- |
| T1 | 4.55 |
| T2 | 0.55 |
| T3 | 2.65 |
| T4 | 2.65 |
| T5 | 2.65 |
| T6 | 0.65 |
| T7 | 0.7 |
| T8 | 0.75 |
| T9 | 0.8 |
| T10 | 1 |
| T11 | 1.05 |
| T12 | 1.25 |
| L1 | 1.2 |
| L2 | 1.2 |
| L3 | 1.15 |
| L4 | 1.3 |
| L5 | 1.3 |

**Additional sagittal profiles in Group I and Group II and results of the FEA**

To evaluate the role of changes in the rods’ curve in the 3D deformation of the rods, additional *S shaped* curves were created by interpolating between the sagittal curve types in Group I (i.e., Types 2 and 4) and by interpolating between the sagittal curve types in Group II (i.e., Types 1, 3, and 5). The curves of the three non-scoliotic adolescents were also modeled separately, as opposed the average curve that was reported in the manuscript.

Figures 1S-A to 1S-C show the sagittal curve of these interpolated curves.

The results of the FEA and the 3D deformations of these curves are shown in figure 2S-A for Group I curves (Model 1 to Model 5- sagittal curves in Figure 1S-A), figure 2S-B for Group II curves (Model 6 to Model 11- sagittal curves in Figure 1S-B), and for the non-scoliotic spines (Model 12 to Model 14- sagittal curves in Figure 1S-C).

Finally the axial projection of the normalized deformation in Group I curves (Model 1 to Model 5), Group II curves (Model 6 to Model 11), and the three non-scoliotic adolescents (Model 12 to Model 14). All the curves in Groups I (Types 2 and 4) and the interpolated curves between the Types 2 and 4 (Figure 1S-A) showed a looped shaped axial deformation (Figure 3S-A) whereas all the curves in Groups II (Types 1, 3 and 5) and the interpolated curves between the Types 1, 3, and 5 (Figure 1S-B) showed a lemniscate shaped axial deformation (Figure 3S-B)

Figure 1S- The interpolated sagittal profiles between the Types 2 and 4 (Group I clusters) and between Types 1, 3, and 5 (Group II clusters) and the normalized *S shape*d curves of the three non-scoliotic spines are shown in Figure 1S-C. The finite element model was generated for all these sagittal profiles. A total of 14 models were created.


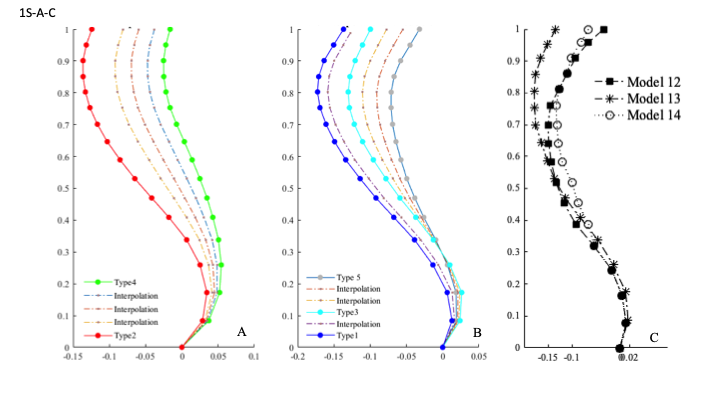


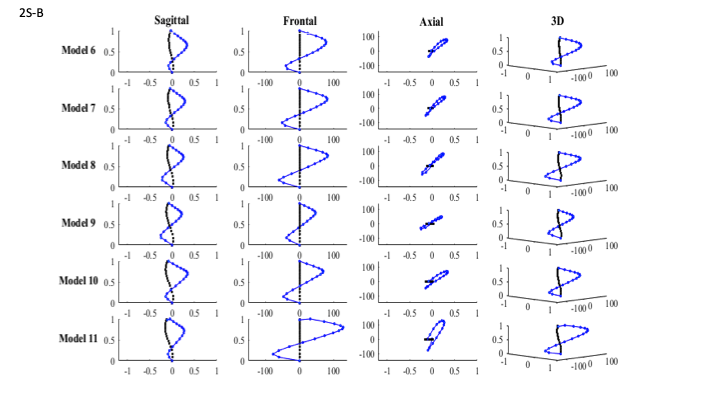
Figure 2S- 3D deformation of the sagittal curve types in figure 1-S: A) Model 1 to Model 5: the S shaped curves in Group I. B) Model 6 to model 11 the S shaped curves in Group II. C) The 3D deformation of the three non-scoliotic curves (Model 12-Model 14).
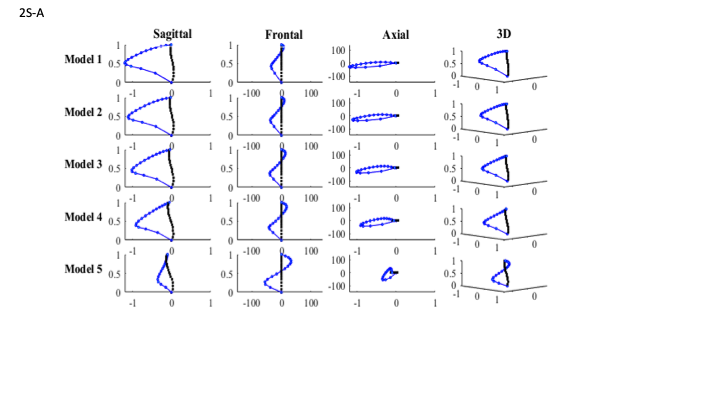


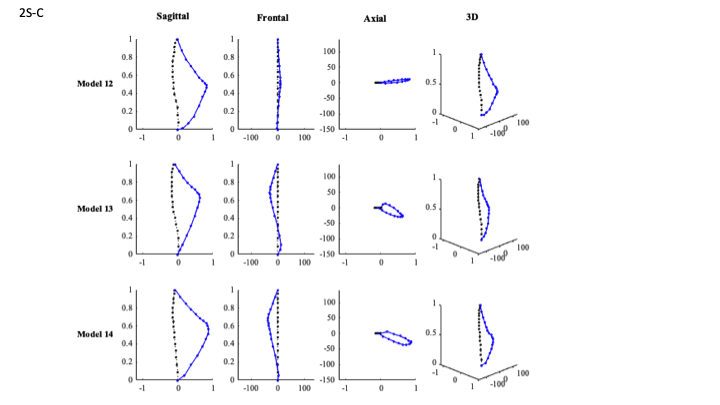


Figure 3S- The axial projection of the 3D deformation for A- Group I models (models 1-5), B) Group II models (models 6-11), and C) non-scoliotic models (Models 12-14).

References

1. Pearsall DJ, Reid JG, Livingston LA. Segmental inertial parameters of the human trunk as determined from computed tomography. *Ann Biomed Eng.* 1996;24(2):198-210.

2. Liu YK, Laborde JM, Van Buskirk WC. Inertial properties of a segmented cadaver trunk: their implications in acceleration injuries. *Aerosp Med.* 1971;42(6):650-657.

3. Clin J, Aubin CE, Lalonde N, Parent S, Labelle H. A new method to include the gravitational forces in a finite element model of the scoliotic spine. *Med Biol Eng Comput.* 2011;49(8):967-977.

4. Pasha S, Aubin CE, Labelle H, Parent S, Mac-Thiong JM. Biomechanical analysis of spino-pelvic parameters in adolescent idiopathic scoliosis after spinal instrumentation and fusion: a case study. *Stud Health Technol Inform.* 2012;176:125-128.
